# Bis[1-meth­oxy-2,2,2-tris­(pyrazol-1-yl-κ*N* ^2^)ethane]­nickel(II) bis­(tri­fluoro­methane­sulfonate) methanol disolvate

**DOI:** 10.1107/S1600536813024653

**Published:** 2013-09-12

**Authors:** Ganna Lyubartseva, Sean Parkin, Uma Prasad Mallik

**Affiliations:** a Department of Biochemistry, Chemistry and Physics, Southern Arkansas University, Magnolia, AR 71753, USA; b Department of Chemistry, University of Kentucky, Lexington, KY 40506, USA

## Abstract

In the title salt, [Ni(C_12_H_14_N_6_O)_2_](CF_3_SO_3_)_2_·2CH_3_OH, the Ni^II^ ion is coordinated by six N atoms from two tridentate 1-meth­oxy-2,2,2-tris­(pyrazol-1-yl)ethane ligands in a distorted octa­hedral geometry. The Ni^II^ ion is situated on an inversion centre. The Ni—N distances range from 2.0589 (19) to 2.0757 (19) Å, intra-ligand N—Ni—N angles range from 84.50 (8) to 85.15 (8)°, and adjacent inter-ligand N—Ni—N angles range between 94.85 (8) and 95.50 (8)°. In the crystal, O—H⋯O hydrogen bonds between methanol solvent mol­ecules and tri­fluoro­methane­sulfonate anions are observed.

## Related literature

Pyrazole-based tridentate ligands are drawing attention because of their topology and the nature of the donor atoms, see: Paulo *et al.* (2004 ▶); Bigmore *et al.* (2005 ▶). For the synthesis of the ligand, see: Maria *et al.* (2007 ▶). The compound reported here was prepared as part of our ongoing research effort to study nitro­gen donor tridentate scorpionate ligands coordinating to nickel, see: Lyubartseva *et al.* (2011 ▶, 2012 ▶); Lyubartseva & Parkin (2009 ▶).
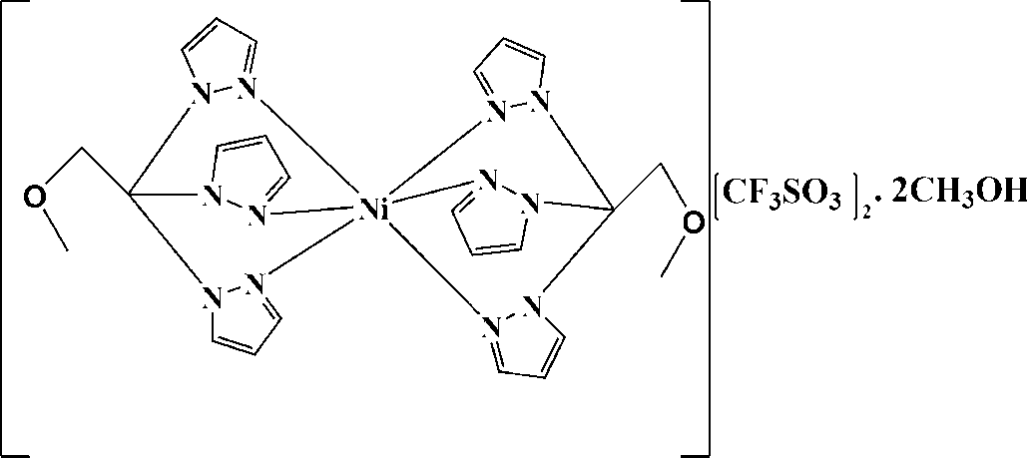


## Experimental

### 

#### Crystal data

[Ni(C_12_H_14_N_6_O)_2_](CF_3_O_3_S)_2_·2CH_4_O
*M* *_r_* = 937.52
Triclinic, 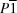

*a* = 9.0025 (2) Å
*b* = 9.5921 (2) Å
*c* = 11.9914 (2) Å
α = 105.2683 (8)°
β = 103.4796 (8)°
γ = 102.2596 (8)°
*V* = 929.15 (3) Å^3^
*Z* = 1
Mo *K*α radiation
μ = 0.74 mm^−1^
*T* = 90 K
0.19 × 0.18 × 0.15 mm

#### Data collection

Nonius KappaCCD diffractometer
Absorption correction: multi-scan (*SADABS*; Sheldrick, 2008*a* ▶) *T*_min_ = 0.753, *T*_max_ = 0.898
22611 measured reflections
4271 independent reflections
3292 reflections with *I* > 2σ(*I*)
*R*_int_ = 0.040

#### Refinement

*R*[*F* ^2^ > 2σ(*F* ^2^)] = 0.043
*wR*(*F* ^2^) = 0.105
*S* = 1.10
4271 reflections
271 parameters
H-atom parameters constrained
Δρ_max_ = 0.45 e Å^−3^
Δρ_min_ = −0.48 e Å^−3^

### 

Data collection: *COLLECT* (Nonius, 1998 ▶); cell refinement: *SCALEPACK* (Otwinowski & Minor, 1997 ▶); data reduction: *DENZO-SMN* (Otwinowski & Minor, 1997 ▶); program(s) used to solve structure: *SHELXS97* (Sheldrick, 2008*b* ▶); program(s) used to refine structure: *SHELXL2013* (Sheldrick, 2008*b* ▶); molecular graphics: *XP* in *SHELXTL* (Sheldrick, 2008*b* ▶); software used to prepare material for publication: *SHELXL2013*.

## Supplementary Material

Crystal structure: contains datablock(s) global, I. DOI: 10.1107/S1600536813024653/lh5647sup1.cif

Structure factors: contains datablock(s) I. DOI: 10.1107/S1600536813024653/lh5647Isup2.hkl

Additional supplementary materials:  crystallographic information; 3D view; checkCIF report

## Figures and Tables

**Table 1:** Hydrogen-bond geometry (Å, °)

*D*—H⋯*A*	*D*—H	H⋯*A*	*D*⋯*A*	*D*—H⋯*A*
O1*S*—H1*S*⋯O2*A*	0.84	1.96	2.782 (3)	168

**Fig. 1.**
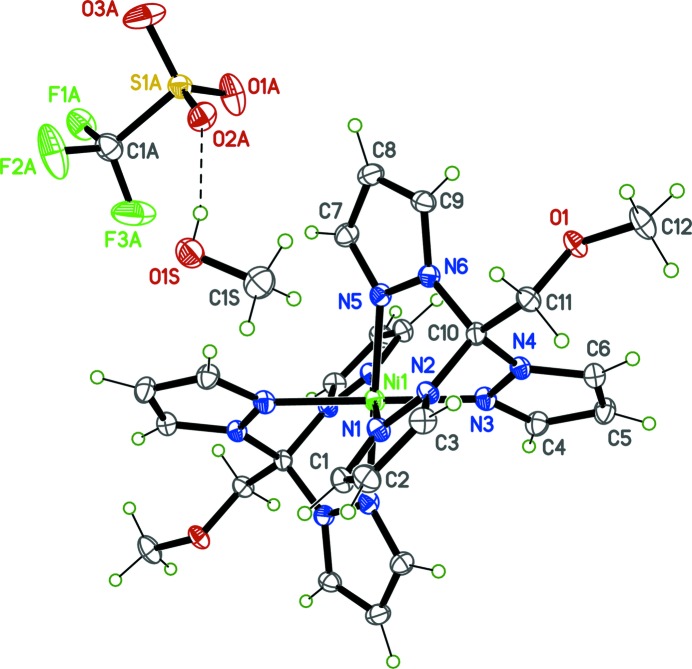
The title compound with displacement ellipsoids drawn at the 50% probability level. Unlabeled atoms are related by the symmetry operator (-x+1, -y+1, -z+1). Only the symmetry unique anion and solvent molecule are shown.

## supplementary crystallographic information

### 1. Comment

In an attempt to prepare mononuclear [*L*_2_Ni^II^]^+2^, where *L* is 1-methoxy-2,2,2-tris(pyrazol-1-yl)ethane, a tridentate neutral nitrogen donor ligand, we isolated the major product [Ni(C_12_H_14_N_60_)_2_][CF_3_SO_3_]_2_·2CH_3_OH as pink triclinic crystals. In the crystal, the nickel ion is coordinated by six N atoms from the two tridentate tpmOMe ligands (average Ni—N distance = 2.0653 Å) in a distorted octahedral geometry. The Ni atom is situated on an inversion centre. The average N—Ni—N angle between adjacent pyrazole-ring-coordinated N atoms is 84.81° for the six acute angles and 95.19° for the six obtuse angles. Intramolecular O—H···O hydrogen bonds are present between methanol solvent molecules and trifluoromethanesulfonate anions.

### 2. Experimental

The 1-methoxy-2,2,2-tris(pyrazol-1-yl)ethane ligand was synthesized according to the previously published procedure of Maria *et al.* (2007). Nickel trifluoromethanesulfonate was used as received. Ni(OTf)_2_(358 mg, 1 mmol) was dissolved in 40 ml me thanol. 1-Methoxy-2,2,2-tris(pyrazol-1-yl)ethane (258 mg, 1 mmol) was dissolved in 25 ml me thanol. The ligand solution was added dropwise to metal solution with moderate stirring. Once the addition was complete, the resulting solution was filtered and solvent was slowly evaporated in air. Pink crystals were obtained after 2 weeks (343 mg, 73.2% yield). Elemental analysis, calculated for C_28_H_36_N_12_NiO_10_F_6_S_2_: C 35.87, H 3.87, N 17.93; found C 35.69, H 3.64, N 18.02. IR (cm^-1^): 3625,3483,3146,2921,1616,1522,1421,1388,1341,1324,1254,1232,1199,1167, 1106,1071,1059, 1028,1011,973,920,855,757,673,653,636,603,573,517.

### 3. Refinement

H atoms were found in difference Fourier maps and subsequently placed at idealized positions with constrained distances of 0.98 Å (RCH_3_), 1.00 Å (*R*_3_CH), 0.95 Å (C_sp2_H), 0.84 Å (O—H), and with *U*_iso_(H) values set to either 1.2*U*_eq_ or 1.5*U*_eq_ (RCH_3_, OH) of the attached atom.

### Crystal data

**Table:**

[Ni(C_12_H_14_N_6_O)_2_](CF_3_O_3_S)_2_·2CH_4_O	*Z* = 1
*M**_r_* = 937.52	*F*(000) = 482
Triclinic, *P*1	*D*_x_ = 1.675 Mg m^−^^3^
*a* = 9.0025 (2) Å	Mo *K*α radiation, λ = 0.71073 Å
*b* = 9.5921 (2) Å	Cell parameters from 4236 reflections
*c* = 11.9914 (2) Å	θ = 1.0–27.5°
α = 105.2683 (8)°	µ = 0.74 mm^−^^1^
β = 103.4796 (8)°	*T* = 90 K
γ = 102.2596 (8)°	Block, pink
*V* = 929.15 (3) Å^3^	0.19 × 0.18 × 0.15 mm

### Data collection

**Table:**

Nonius KappaCCD diffractometer	4271 independent reflections
Radiation source: fine-focus sealed-tube	3292 reflections with *I* > 2σ(*I*)
Detector resolution: 9.1 pixels mm^-1^	*R*_int_ = 0.040
φ and ω scans at fixed χ = 55°	θ_max_ = 27.5°, θ_min_ = 1.9°
Absorption correction: multi-scan (*SADABS*; Sheldrick, 2008*a*)	*h* = −11→11
*T*_min_ = 0.753, *T*_max_ = 0.898	*k* = −12→12
22611 measured reflections	*l* = −15→15

### Refinement

**Table:**

Refinement on *F*^2^	Primary atom site location: structure-invariant direct methods
Least-squares matrix: full	Secondary atom site location: difference Fourier map
*R*[*F*^2^ > 2σ(*F*^2^)] = 0.043	Hydrogen site location: inferred from neighbouring sites
*wR*(*F*^2^) = 0.105	H-atom parameters constrained
*S* = 1.10	*w* = 1/[σ^2^(*F*_o_^2^) + (0.0411*P*)^2^ + 1.0985*P*] where *P* = (*F*_o_^2^ + 2*F*_c_^2^)/3
4271 reflections	(Δ/σ)_max_ < 0.001
271 parameters	Δρ_max_ = 0.45 e Å^−^^3^
0 restraints	Δρ_min_ = −0.48 e Å^−^^3^

### Special details

**Table:**

Geometry. All e.s.d.'s (except the e.s.d. in the dihedral angle between two l.s. planes) are estimated using the full covariance matrix. The cell e.s.d.'s are taken into account individually in the estimation of e.s.d.'s in distances, angles and torsion angles; correlations between e.s.d.'s in cell parameters are only used when they are defined by crystal symmetry. An approximate (isotropic) treatment of cell e.s.d.'s is used for estimating e.s.d.'s involving l.s. planes.

### Fractional atomic coordinates and isotropic or equivalent isotropic displacement parameters (Å^2^)

**Table:**

	*x*	*y*	*z*	*U*_iso_*/*U*_eq_	
Ni1	0.5000	0.5000	0.5000	0.01541 (12)	
N1	0.5656 (2)	0.7100 (2)	0.63001 (18)	0.0170 (4)	
N2	0.7195 (2)	0.7717 (2)	0.70477 (17)	0.0146 (4)	
C1	0.4910 (3)	0.8115 (3)	0.6624 (2)	0.0183 (5)	
H1	0.3814	0.7993	0.6255	0.022*	
C2	0.5939 (3)	0.9387 (3)	0.7576 (2)	0.0215 (5)	
H2	0.5688	1.0264	0.7968	0.026*	
C3	0.7388 (3)	0.9105 (3)	0.7828 (2)	0.0188 (5)	
H3	0.8347	0.9757	0.8433	0.023*	
N3	0.6331 (2)	0.4422 (2)	0.63761 (18)	0.0166 (4)	
N4	0.7765 (2)	0.5424 (2)	0.71273 (17)	0.0148 (4)	
C4	0.6123 (3)	0.3275 (3)	0.6808 (2)	0.0199 (5)	
H4	0.5224	0.2401	0.6468	0.024*	
C5	0.7402 (3)	0.3530 (3)	0.7828 (2)	0.0211 (5)	
H5	0.7536	0.2881	0.8296	0.025*	
C6	0.8421 (3)	0.4903 (3)	0.8014 (2)	0.0187 (5)	
H6	0.9405	0.5400	0.8646	0.022*	
N5	0.7151 (2)	0.5746 (2)	0.47104 (17)	0.0162 (4)	
N6	0.8478 (2)	0.6538 (2)	0.56911 (17)	0.0156 (4)	
C7	0.7638 (3)	0.5711 (3)	0.3745 (2)	0.0195 (5)	
H7	0.6968	0.5233	0.2929	0.023*	
C8	0.9267 (3)	0.6469 (3)	0.4085 (2)	0.0210 (5)	
H8	0.9895	0.6592	0.3563	0.025*	
C9	0.9763 (3)	0.6993 (3)	0.5324 (2)	0.0185 (5)	
H9	1.0812	0.7569	0.5835	0.022*	
C10	0.8346 (3)	0.6854 (3)	0.6921 (2)	0.0150 (5)	
C11	0.9962 (3)	0.7798 (3)	0.7859 (2)	0.0170 (5)	
H11A	1.0337	0.8761	0.7712	0.020*	
H11B	0.9858	0.8034	0.8688	0.020*	
O1	1.10620 (19)	0.69583 (18)	0.77534 (15)	0.0191 (4)	
C12	1.2450 (3)	0.7555 (3)	0.8798 (2)	0.0267 (6)	
H12A	1.3066	0.8544	0.8824	0.040*	
H12B	1.3111	0.6864	0.8752	0.040*	
H12C	1.2124	0.7667	0.9534	0.040*	
S1A	0.85393 (7)	0.75255 (7)	0.11470 (5)	0.02007 (15)	
O1A	0.8467 (3)	0.6007 (2)	0.11115 (19)	0.0381 (5)	
O2A	0.8839 (2)	0.8585 (2)	0.23378 (15)	0.0252 (4)	
O3A	0.9434 (2)	0.8109 (3)	0.04420 (17)	0.0406 (6)	
C1A	0.6486 (3)	0.7358 (3)	0.0358 (2)	0.0261 (6)	
F1A	0.60356 (18)	0.64929 (18)	−0.08066 (13)	0.0306 (4)	
F2A	0.6306 (3)	0.8709 (2)	0.03702 (18)	0.0555 (6)	
F3A	0.5478 (2)	0.6780 (2)	0.08729 (17)	0.0503 (5)	
O1S	0.7190 (2)	0.9839 (2)	0.38362 (17)	0.0275 (4)	
H1S	0.7699	0.9374	0.3445	0.041*	
C1S	0.7689 (3)	0.9892 (3)	0.5057 (3)	0.0286 (6)	
H1S1	0.8850	1.0335	0.5393	0.043*	
H1S2	0.7396	0.8866	0.5096	0.043*	
H1S3	0.7167	1.0512	0.5529	0.043*	

### Atomic displacement parameters (Å^2^)

**Table:**

	*U*^11^	*U*^22^	*U*^33^	*U*^12^	*U*^13^	*U*^23^
Ni1	0.0140 (2)	0.0148 (2)	0.0151 (2)	0.00295 (17)	0.00210 (17)	0.00417 (17)
N1	0.0132 (10)	0.0165 (10)	0.0173 (10)	0.0023 (8)	0.0016 (8)	0.0039 (8)
N2	0.0119 (9)	0.0146 (9)	0.0155 (10)	0.0033 (8)	0.0024 (8)	0.0039 (8)
C1	0.0184 (12)	0.0176 (12)	0.0196 (12)	0.0078 (10)	0.0047 (10)	0.0064 (10)
C2	0.0225 (13)	0.0173 (12)	0.0246 (13)	0.0075 (10)	0.0075 (10)	0.0051 (10)
C3	0.0188 (12)	0.0154 (12)	0.0178 (12)	0.0023 (9)	0.0042 (10)	0.0020 (9)
N3	0.0129 (9)	0.0153 (10)	0.0167 (10)	0.0006 (8)	0.0009 (8)	0.0036 (8)
N4	0.0136 (9)	0.0137 (9)	0.0150 (10)	0.0030 (8)	0.0023 (8)	0.0039 (8)
C4	0.0207 (12)	0.0174 (12)	0.0212 (12)	0.0035 (10)	0.0058 (10)	0.0079 (10)
C5	0.0229 (13)	0.0207 (12)	0.0210 (13)	0.0056 (10)	0.0053 (10)	0.0109 (10)
C6	0.0185 (12)	0.0198 (12)	0.0181 (12)	0.0067 (10)	0.0039 (10)	0.0073 (10)
N5	0.0143 (10)	0.0168 (10)	0.0130 (10)	0.0014 (8)	0.0006 (8)	0.0031 (8)
N6	0.0144 (10)	0.0172 (10)	0.0127 (9)	0.0035 (8)	0.0026 (8)	0.0032 (8)
C7	0.0209 (12)	0.0199 (12)	0.0165 (12)	0.0047 (10)	0.0058 (10)	0.0050 (10)
C8	0.0234 (13)	0.0219 (13)	0.0222 (13)	0.0077 (10)	0.0122 (10)	0.0092 (10)
C9	0.0150 (11)	0.0188 (12)	0.0223 (13)	0.0046 (9)	0.0068 (10)	0.0072 (10)
C10	0.0144 (11)	0.0154 (11)	0.0147 (11)	0.0041 (9)	0.0028 (9)	0.0059 (9)
C11	0.0140 (11)	0.0160 (11)	0.0168 (12)	0.0028 (9)	0.0021 (9)	0.0024 (9)
O1	0.0132 (8)	0.0203 (9)	0.0198 (9)	0.0063 (7)	0.0003 (7)	0.0034 (7)
C12	0.0170 (13)	0.0287 (14)	0.0262 (14)	0.0077 (11)	−0.0035 (11)	0.0040 (11)
S1A	0.0172 (3)	0.0233 (3)	0.0172 (3)	0.0048 (2)	0.0039 (2)	0.0049 (2)
O1A	0.0420 (12)	0.0284 (11)	0.0354 (12)	0.0180 (9)	−0.0050 (9)	0.0053 (9)
O2A	0.0288 (10)	0.0268 (10)	0.0166 (9)	0.0059 (8)	0.0059 (8)	0.0043 (7)
O3A	0.0269 (11)	0.0574 (14)	0.0206 (10)	−0.0127 (10)	0.0082 (8)	0.0045 (10)
C1A	0.0256 (14)	0.0271 (14)	0.0246 (14)	0.0103 (11)	0.0070 (11)	0.0053 (11)
F1A	0.0256 (8)	0.0333 (9)	0.0226 (8)	0.0081 (7)	−0.0013 (6)	0.0008 (7)
F2A	0.0671 (14)	0.0359 (10)	0.0487 (12)	0.0332 (10)	−0.0113 (10)	0.0014 (9)
F3A	0.0248 (9)	0.0771 (14)	0.0403 (11)	0.0038 (9)	0.0153 (8)	0.0096 (10)
O1S	0.0268 (10)	0.0322 (11)	0.0262 (10)	0.0138 (8)	0.0083 (8)	0.0096 (8)
C1S	0.0317 (15)	0.0238 (14)	0.0305 (15)	0.0068 (12)	0.0099 (12)	0.0098 (12)

### Geometric parameters (Å, º)

**Table:**

Ni1—N5^i^	2.0589 (19)	N6—C10	1.464 (3)
Ni1—N5	2.059 (2)	C7—C8	1.399 (3)
Ni1—N1^i^	2.0611 (19)	C7—H7	0.9500
Ni1—N1	2.0611 (19)	C8—C9	1.364 (3)
Ni1—N3^i^	2.0757 (19)	C8—H8	0.9500
Ni1—N3	2.0757 (19)	C9—H9	0.9500
N1—C1	1.323 (3)	C10—C11	1.531 (3)
N1—N2	1.366 (3)	C11—O1	1.410 (3)
N2—C3	1.359 (3)	C11—H11A	0.9900
N2—C10	1.468 (3)	C11—H11B	0.9900
C1—C2	1.394 (3)	O1—C12	1.431 (3)
C1—H1	0.9500	C12—H12A	0.9800
C2—C3	1.369 (3)	C12—H12B	0.9800
C2—H2	0.9500	C12—H12C	0.9800
C3—H3	0.9500	S1A—O3A	1.432 (2)
N3—C4	1.331 (3)	S1A—O1A	1.433 (2)
N3—N4	1.369 (3)	S1A—O2A	1.4437 (18)
N4—C6	1.357 (3)	S1A—C1A	1.821 (3)
N4—C10	1.466 (3)	C1A—F3A	1.318 (3)
C4—C5	1.396 (3)	C1A—F2A	1.336 (3)
C4—H4	0.9500	C1A—F1A	1.337 (3)
C5—C6	1.364 (4)	O1S—C1S	1.411 (3)
C5—H5	0.9500	O1S—H1S	0.8400
C6—H6	0.9500	C1S—H1S1	0.9800
N5—C7	1.324 (3)	C1S—H1S2	0.9800
N5—N6	1.370 (3)	C1S—H1S3	0.9800
N6—C9	1.359 (3)		
			
N5^i^—Ni1—N5	180.0	C9—N6—C10	129.4 (2)
N5^i^—Ni1—N1^i^	85.15 (8)	N5—N6—C10	119.86 (18)
N5—Ni1—N1^i^	94.85 (8)	N5—C7—C8	111.1 (2)
N5^i^—Ni1—N1	94.85 (8)	N5—C7—H7	124.4
N5—Ni1—N1	85.15 (8)	C8—C7—H7	124.4
N1^i^—Ni1—N1	180.00 (11)	C9—C8—C7	105.6 (2)
N5^i^—Ni1—N3^i^	84.78 (8)	C9—C8—H8	127.2
N5—Ni1—N3^i^	95.22 (8)	C7—C8—H8	127.2
N1^i^—Ni1—N3^i^	84.50 (8)	N6—C9—C8	107.2 (2)
N1—Ni1—N3^i^	95.50 (8)	N6—C9—H9	126.4
N5^i^—Ni1—N3	95.22 (8)	C8—C9—H9	126.4
N5—Ni1—N3	84.78 (8)	N6—C10—N4	109.33 (18)
N1^i^—Ni1—N3	95.50 (8)	N6—C10—N2	109.35 (18)
N1—Ni1—N3	84.50 (8)	N4—C10—N2	108.62 (18)
N3^i^—Ni1—N3	180.00 (8)	N6—C10—C11	110.37 (19)
C1—N1—N2	105.50 (19)	N4—C10—C11	110.87 (18)
C1—N1—Ni1	134.77 (17)	N2—C10—C11	108.27 (18)
N2—N1—Ni1	119.73 (14)	O1—C11—C10	108.34 (18)
C3—N2—N1	110.70 (19)	O1—C11—H11A	110.0
C3—N2—C10	130.25 (19)	C10—C11—H11A	110.0
N1—N2—C10	119.05 (18)	O1—C11—H11B	110.0
N1—C1—C2	111.3 (2)	C10—C11—H11B	110.0
N1—C1—H1	124.4	H11A—C11—H11B	108.4
C2—C1—H1	124.4	C11—O1—C12	111.62 (18)
C3—C2—C1	105.5 (2)	O1—C12—H12A	109.5
C3—C2—H2	127.2	O1—C12—H12B	109.5
C1—C2—H2	127.2	H12A—C12—H12B	109.5
N2—C3—C2	107.0 (2)	O1—C12—H12C	109.5
N2—C3—H3	126.5	H12A—C12—H12C	109.5
C2—C3—H3	126.5	H12B—C12—H12C	109.5
C4—N3—N4	105.24 (19)	O3A—S1A—O1A	115.91 (14)
C4—N3—Ni1	135.74 (16)	O3A—S1A—O2A	113.55 (12)
N4—N3—Ni1	118.90 (14)	O1A—S1A—O2A	115.10 (12)
C6—N4—N3	110.88 (19)	O3A—S1A—C1A	103.65 (13)
C6—N4—C10	129.58 (19)	O1A—S1A—C1A	102.79 (12)
N3—N4—C10	119.51 (18)	O2A—S1A—C1A	103.55 (12)
N3—C4—C5	110.9 (2)	F3A—C1A—F2A	107.0 (2)
N3—C4—H4	124.6	F3A—C1A—F1A	108.1 (2)
C5—C4—H4	124.6	F2A—C1A—F1A	106.9 (2)
C6—C5—C4	105.9 (2)	F3A—C1A—S1A	111.68 (19)
C6—C5—H5	127.0	F2A—C1A—S1A	111.05 (19)
C4—C5—H5	127.0	F1A—C1A—S1A	111.86 (18)
N4—C6—C5	107.1 (2)	C1S—O1S—H1S	109.5
N4—C6—H6	126.5	O1S—C1S—H1S1	109.5
C5—C6—H6	126.5	O1S—C1S—H1S2	109.5
C7—N5—N6	105.39 (19)	H1S1—C1S—H1S2	109.5
C7—N5—Ni1	135.53 (16)	O1S—C1S—H1S3	109.5
N6—N5—Ni1	119.02 (14)	H1S1—C1S—H1S3	109.5
C9—N6—N5	110.65 (18)	H1S2—C1S—H1S3	109.5
			
C1—N1—N2—C3	0.1 (3)	N5—N6—C10—N4	59.9 (2)
Ni1—N1—N2—C3	179.31 (15)	C9—N6—C10—N2	117.0 (2)
C1—N1—N2—C10	179.7 (2)	N5—N6—C10—N2	−58.9 (3)
Ni1—N1—N2—C10	−1.1 (3)	C9—N6—C10—C11	−2.0 (3)
N2—N1—C1—C2	−0.2 (3)	N5—N6—C10—C11	−177.94 (18)
Ni1—N1—C1—C2	−179.22 (17)	C6—N4—C10—N6	124.2 (2)
N1—C1—C2—C3	0.2 (3)	N3—N4—C10—N6	−58.1 (3)
N1—N2—C3—C2	0.0 (3)	C6—N4—C10—N2	−116.5 (2)
C10—N2—C3—C2	−179.5 (2)	N3—N4—C10—N2	61.2 (3)
C1—C2—C3—N2	−0.1 (3)	C6—N4—C10—C11	2.3 (3)
C4—N3—N4—C6	−0.4 (3)	N3—N4—C10—C11	−179.97 (19)
Ni1—N3—N4—C6	176.26 (15)	C3—N2—C10—N6	−120.8 (2)
C4—N3—N4—C10	−178.5 (2)	N1—N2—C10—N6	59.7 (3)
Ni1—N3—N4—C10	−1.8 (3)	C3—N2—C10—N4	120.0 (2)
N4—N3—C4—C5	0.1 (3)	N1—N2—C10—N4	−59.5 (2)
Ni1—N3—C4—C5	−175.75 (18)	C3—N2—C10—C11	−0.5 (3)
N3—C4—C5—C6	0.2 (3)	N1—N2—C10—C11	−179.99 (19)
N3—N4—C6—C5	0.6 (3)	N6—C10—C11—O1	−61.4 (2)
C10—N4—C6—C5	178.4 (2)	N4—C10—C11—O1	59.9 (2)
C4—C5—C6—N4	−0.5 (3)	N2—C10—C11—O1	178.97 (18)
C7—N5—N6—C9	0.5 (3)	C10—C11—O1—C12	−163.6 (2)
Ni1—N5—N6—C9	−177.23 (15)	O3A—S1A—C1A—F3A	177.87 (19)
C7—N5—N6—C10	177.1 (2)	O1A—S1A—C1A—F3A	56.8 (2)
Ni1—N5—N6—C10	−0.6 (3)	O2A—S1A—C1A—F3A	−63.4 (2)
N6—N5—C7—C8	0.1 (3)	O3A—S1A—C1A—F2A	−62.8 (2)
Ni1—N5—C7—C8	177.22 (17)	O1A—S1A—C1A—F2A	176.2 (2)
N5—C7—C8—C9	−0.6 (3)	O2A—S1A—C1A—F2A	56.0 (2)
N5—N6—C9—C8	−0.9 (3)	O3A—S1A—C1A—F1A	56.6 (2)
C10—N6—C9—C8	−177.1 (2)	O1A—S1A—C1A—F1A	−64.5 (2)
C7—C8—C9—N6	0.9 (3)	O2A—S1A—C1A—F1A	175.33 (18)
C9—N6—C10—N4	−124.2 (2)		

Symmetry code: (i) −*x*+1, −*y*+1, −*z*+1.

### Hydrogen-bond geometry (Å, º)

**Table:**

*D*—H···*A*	*D*—H	H···*A*	*D*···*A*	*D*—H···*A*
O1*S*—H1*S*···O2*A*	0.84	1.96	2.782 (3)	168
